# RhoA-ROCK Signaling as a Therapeutic Target in Traumatic Brain Injury

**DOI:** 10.3390/cells9010245

**Published:** 2020-01-18

**Authors:** Shalaka Mulherkar, Kimberley F. Tolias

**Affiliations:** 1 Department of Neuroscience, Baylor College of Medicine, Houston, TX 77030, USA; mulherka@bcm.edu; 2 Verna and Marrs McLean Department of Biochemistry and Molecular Biology, Baylor College of Medicine, Houston, TX 77030, USA

**Keywords:** Rho GTPases, RhoA, ROCK, TBI, synapse, dendritic spine, actin, CNS injury

## Abstract

Traumatic brain injury (TBI) is a leading cause of death and disability worldwide. TBIs, which range in severity from mild to severe, occur when a traumatic event, such as a fall, a traffic accident, or a blow, causes the brain to move rapidly within the skull, resulting in damage. Long-term consequences of TBI can include motor and cognitive deficits and emotional disturbances that result in a reduced quality of life and work productivity. Recovery from TBI can be challenging due to a lack of effective treatment options for repairing TBI-induced neural damage and alleviating functional impairments. Central nervous system (CNS) injury and disease are known to induce the activation of the small GTPase RhoA and its downstream effector Rho kinase (ROCK). Activation of this signaling pathway promotes cell death and the retraction and loss of neural processes and synapses, which mediate information flow and storage in the brain. Thus, inhibiting RhoA-ROCK signaling has emerged as a promising approach for treating CNS disorders. In this review, we discuss targeting the RhoA-ROCK pathway as a therapeutic strategy for treating TBI and summarize the recent advances in the development of RhoA-ROCK inhibitors.

## 1. Traumatic Brain Injury

Traumatic brain injury (TBI) is a major health problem that affects approximately 2.8 million people in the United States each year and contributes to around 30% of all injury-related deaths [1]. TBI is defined as a blow, jolt, or penetrating head injury that disrupts normal brain function. TBI severity ranges from mild to severe, with moderate and severe TBIs (as well as repeated mild TBIs) inducing neural damage, cell death, and disruption of neural circuits that result in long-term motor, cognitive and behavioral deficits. In addition to lifelong impairments in learning, memory, and attention, TBI survivors often suffer from depression, anxiety, and personality changes [2,3]. These issues not only affect individuals but also have adverse effects on family members and communities. The leading causes of TBIs include falls (which particularly impact children and older adults), being struck by or against an object, traffic accidents, and assaults [1]. Sports-related TBIs, which are common in adolescents and young adults and often go unreported, pose a particular problem since recurrent head injuries increase the risk of developing a progressive neurodegenerative disorder known as chronic traumatic encephalopathy (CTE) [4,5]. Military veterans who obtained TBIs as a result of blast injuries or gunshot wounds are also at higher risk for developing CTE as well as post-traumatic stress disorder (PTSD) [6,7]. TBI-induced brain damage occurs in two stages. Primary brain injury happens at the time of the initial trauma due to direct mechanical damage, whereas secondary injury develops over time as a consequence of destructive biochemical cascades associated with excitotoxicity, perturbed calcium homeostasis, free radical production, mitochondrial dysfunction, and inflammation [8]. Despite the rapid pace of brain injury research in the last decade, there is a general lack of effective treatments for TBI, and as a result, complete recovery often remains elusive. This review examines the small GTPase RhoA and its major downstream effector Rho-associated kinase (ROCK/ROK/Rho-kinase) as potential promising therapeutic targets to treat TBI. 

## 2. Rho GTPase Signaling

**Figure 1:**
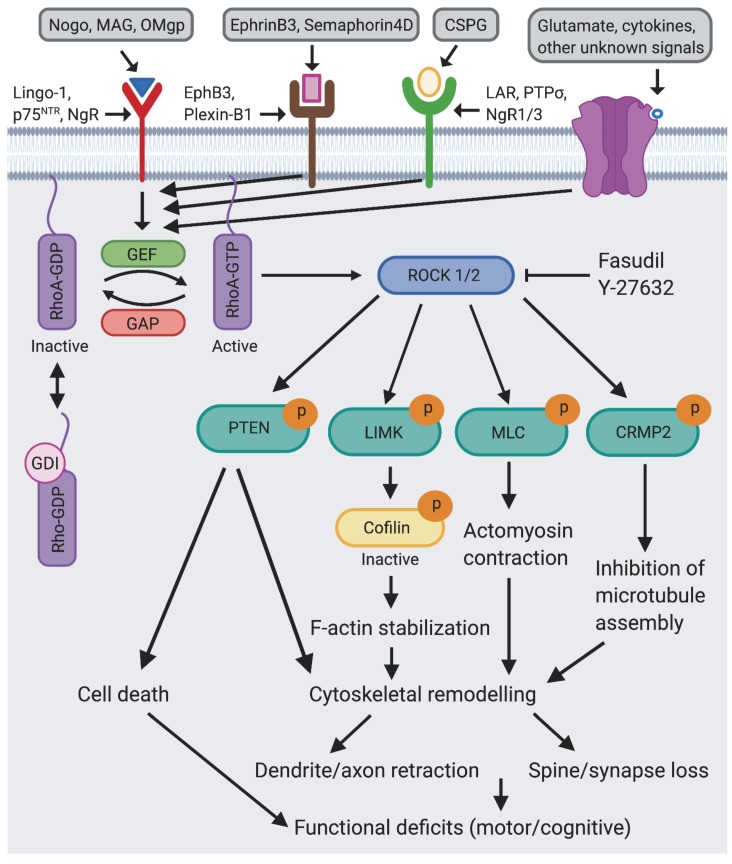
Model for the role of the RhoA-ROCK pathway in the pathogenesis of TBI. The small GTPase RhoA is activated by RhoA-GEFs in response to various extracellular signals triggered by injury. Active GTP-bound RhoA binds to and stimulates the activity of the serine/threonine kinase ROCK1/2. Through phosphorylation of downstream effectors such as PTEN, LIMK, MLC, and CRMP-2, ROCK initiates signaling cascades that induce cytoskeletal remodeling underlying dendrite/axon retraction and synapse/spine loss as well as cell death, which together contribute to functional deficits. Inhibition of ROCK (e.g., Fasudil, Y-27632) or RhoA rescues these TBI-induced deficits. ROCK: Rho Kinase, GEF: guanine nucleotide exchange factor, GAP: GTPase-activating protein, GDI: Guanine nucleotide dissociation inhibitor, PTEN: phosphatase and tensin homolog, LIMK: LIM kinase, MLC: myosin light chain, CRMP2: collapsin response mediator protein 2, MAG: myelin-associated glycoprotein, OMgp: oligodendrocyte-myelin glycoprotein, NgR: nogo receptor, PTPσ: protein tyrosine phosphate σ, NgR1/3: nogo receptor 1 and 3, LAR: leukocyte common antigen-related phosphatase, CSPG: chondroitin sulfate proteoglycans.

Rho-family small GTPases (e.g., RhoA, Rac1, Cdc42) are key regulators of cytoskeletal and cell adhesion dynamics that control a wide range of cellular processes, including morphogenesis, migration, proliferation, and survival [9]. Rho GTPases regulate these processes by functioning as molecular switches, cycling between an active GTP-bound state and an inactive GDP-bound state (Figure 1). This cycling is precisely controlled in space and time by the opposing actions of guanine nucleotide-exchange factors (GEFs), which activate Rho GTPases by facilitating GTP-GDP exchange, and GTPase-activating proteins (GAPs), which inhibit Rho GTPases by catalyzing GTP hydrolysis [10,11]. In their GTP-bound state, Rho GTPases interact with and activate downstream effector proteins, initiating intracellular signaling cascades that affect cell behavior and morphology [9]. A major downstream effector for RhoA is the serine-threonine kinase ROCK1/2 [12,13] (Figure 1). Following RhoA activation, ROCK promotes actomyosin contractile force generation by increasing the phosphorylation of myosin light chain (MLC), a subunit of the actin-based motor protein myosin II [14]. This regulation occurs both directly by phosphorylation of MLC and indirectly by phosphorylation and inhibition of myosin phosphatase [14,15]. RhoA-ROCK signaling also stabilizes actin filaments by inducing the LIM kinase-dependent phosphorylation and inactivation of cofilin, an actin-binding protein that normally mediates actin turnover by severing and disassembling actin filaments [16,17]. Another prominent ROCK substrate is collapsin response mediator protein-2 (CRMP-2), a microtubule-binding protein that stimulates axon growth by promoting microtubule assembly. ROCK-mediated phosphorylation of CRMP-2 inhibits its ability to bind to tubulin and thereby induces growth cone collapse [18]. Additionally, ROCK phosphorylates and stimulates the activity of the dual protein/lipid phosphatase PTEN (phosphatase and tensin homolog), a tumor suppressor that inhibits cell growth and survival [19]. Collectively, these actions of RhoA-ROCK signaling drive actin cytoskeletal remodeling, cell contractility, and cell death (Figure 1).

## 3. Functions of Rho GTPases in the Central Nervous System

Although Rho GTPases play fundamental roles in all cell types, they are particularly important in the CNS. In developing neurons, Rac1 promotes survival, the growth, and branching of axons and dendrites, and the formation and maintenance of dendritic spines, the primary post-synaptic sites of excitatory synapses [20]. In contrast, RhoA typically inhibits these processes, eliciting neuronal death, axonal and dendritic retraction, and spine/synapse loss [20,21]. Rac1 and RhoA are also critical regulators of neuronal migration in the developing CNS, with Rac1 generally promoting and RhoA inhibiting migration [22]. The effects of Rho GTPases on cell migration can also be non-cell autonomous. For example, embryonic deletion of *RHOA* from the mouse cerebral cortex results in subcortical heterotopias and cobblestone lissencephaly, which are caused by migration defects that arise from disorganization of the radial glial scaffold, which normally directs the migration of newborn neurons [23]. Developmental *RHOA* ablation from neuroprogenitor cells also results in decreased progenitor proliferation and a loss of adherens junctions, neuroepithelial organization, and apical-basal polarity [24,25,26,27]. Following development, Rac1 and RhoA continue to play important roles in the structural and functional plasticity of synapses, which is critical for processes such as learning and memory [28]. Moreover, tightly regulated Rho GTPase signaling is necessary for neuronal survival and the proper maintenance of neuronal architecture in the adult brain [11,20].

Given their essential functions in nervous system development, survival, and plasticity, it is not surprising that Rho GTPases also play important roles in CNS disease and injury [29]. Dysregulated Rho GTPase signaling has been implicated in a wide spectrum of neurodevelopmental, neuropsychiatric, and neurodegenerative disorders, including intellectual disorders, autism spectrum disorders, schizophrenia, depression, amyotrophic lateral sclerosis (ALS), Parkinson’s disease (PD), and Alzheimer’s disease (AD) [20,30]. Moreover, activation of Rho GTPases, in particular, RhoA, is thought to be important for mediating the pathogenesis of CNS injury [31]. For instance, in a lateral fluid percussion injury (FPI) model of TBI, RhoA is robustly activated in the ipsilateral rat cortex within 24 h of injury, peaking at day 3 post-injury and remaining high until at least day 7 [32]. Similar high levels of active RhoA are present in the contralateral cortex and hippocampus of injured rats, suggesting that elevated RhoA activity is not restricted to the initial site of impact. Likewise, high levels of RhoA expression and activity are found in the spinal cord neurons and glia of rats and mice following spinal cord injury (SCI), which can last up to three months [33,34]. Moreover, RhoA activation is elevated in the brains of rats following kainic acid-induced seizures [32] and in the eyes of pigs after retinal detachment, which is associated with axonal and synaptic retraction [35,36]. While the precise mechanisms of RhoA activation have not been fully elucidated, these findings suggest that RhoA signaling is a convergence point following CNS injury, irrespective of the original trauma. Notably, consistent with animal studies, immunohistochemistry of post-mortem human brain samples affected by TBI showed upregulation of RhoA and closely-related RhoB within hours of the initial insult and continuing for months after the injury [37]. Given its ability to induce cell death, as well as axon and dendrite retraction and synapse loss [20,21], persistent elevated activity and expression of RhoA after CNS injury could be one of the factors that restrict regeneration and limits complete functional recovery of the injured CNS. If true, inhibiting the RhoA-ROCK signaling pathway may be an effective strategy for enhancing rehabilitation post-injury.

## 4. Targeting the RhoA-ROCK Signaling Pathway in Animal Models of TBI

**Figure 2:**
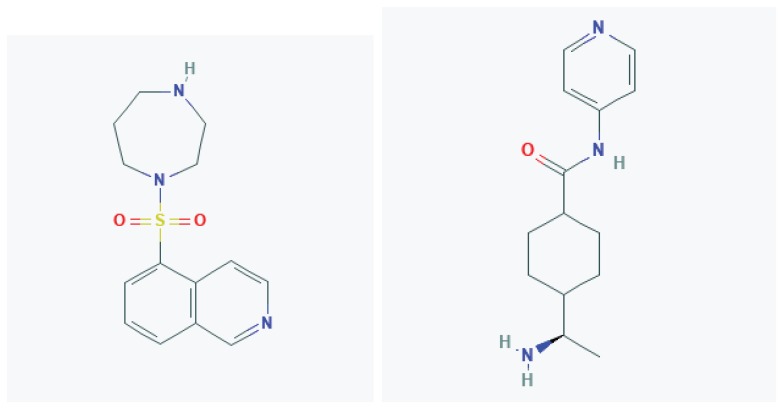
Pharmacological ROCK inhibitors Fasudil and Y-27632.Chemical structures of two widely used ROCK inhibitors, Fasudil and Y-27632. Both ROCK inhibitors have been demonstrated to alleviate functional deficits in mouse models of TBI. Images were taken from the National Center for Biotechnology Information, PubChem Database; Fasudil: https://pubchem.ncbi.nlm.nih.gov/compound/Fasudil; Y-27632: https://pubchem.ncbi.nlm.nih.gov/compound/448042.

Since excessive RhoA-ROCK signaling contributes to the pathophysiology of a wide range of disorders, scientists and clinicians have long been interested in this pathway as a potential therapeutic target [30]. Indeed, accumulating evidence suggests that inhibiting RhoA-ROCK signaling is beneficial for treating conditions such as ocular disease, subarachnoid hemorrhage, SCI, epilepsy, stroke, neuropathic pain, ALS, PD, and AD [38,39,40,41]. To determine whether suppressing RhoA-ROCK signaling enhances recovery from TBI, we and others have investigated the effects of inhibiting RhoA and/or ROCK on mouse models of TBI [42,43]. Bye and colleagues [42] subjected mice to a controlled cortical impact (CCI) model of TBI, and then treated the mice with the ROCK inhibitor Y27632 (Figure 2) for one or four weeks. In both cases, they found that ROCK inhibition improved the motor performance of mice after TBI (i.e., decreased forepaw faults on a horizontal ladder) [42]. Our group addressed this problem using two independent approaches to block RhoA-ROCK signaling, by genetically ablating *RHOA* from postnatal forebrain neurons (RhoA^fl/fl^, CamKIIα-Cre mice) and by treating wild-type mice with the pharmacological ROCK inhibitor fasudil [43] (Figure 2). Consistent with Bye et al. (2016), we found that mice subjected to CCI performed poorly on an accelerating rotarod, displaying transient deficits in motor coordination and balance, and that blocking RhoA-ROCK signaling by either method accelerated the restoration of normal motor function [43]. Likewise, we found that TBI disrupted contextual fear discrimination in mice, impairing their ability to distinguish between a fearful and a non-fearful environment, and that inhibiting RhoA genetically (RhoA^fl/fl^, CamKIIα-Cre mice) or ROCK pharmacologically (fasudil) protected mice against this hippocampal-dependent memory deficit [43]. Together, these findings indicate that blocking RhoA-ROCK signaling alleviates TBI-induced motor and cognitive impairments and thus enhances functional recovery after TBI (Figure 1). Moreover, since genetically ablating *RHOA* and pharmacologically inhibiting ROCK produced similar results, it is likely that ROCK is the primary mediator of TBI-induced deficits rather than other RhoA effector pathways.

How might inhibiting RhoA-ROCK signaling protect against injury-related brain damage and/or promote repair? A major limitation to recovery after CNS injury is the hostile growth environment of the adult CNS that restricts regeneration. Inhibitors of CNS regeneration include myelin-associated inhibitors, glial scar-associated inhibitors, and repulsive axon guidance molecules [44]. Notably, many of these growth inhibitory molecules mediate their effects via activating RhoA-ROCK signaling (Figure 1). For instance, in an injured CNS, myelin-derived axon growth inhibitors such as myelin-associated glycoprotein (MAG), oligodendrocyte myelin glycoprotein (OMgp), and Nogo bind to the Nogo receptor (NgR1), which in cooperation with receptors such as p75^NTR^ and LINGO-1 activates RhoA-ROCK signaling, resulting in growth cone collapse and axon growth inhibition [29,45,46]. Likewise, repulsive axon guidance molecules such as ephrinB3 and semaphorin 4D and glial scar components such as chondroitin sulfate proteoglycans (CSPGs) trigger activation of RhoA-ROCK signaling, resulting in axon outgrowth inhibition [29] (Figure 1). Abrogating RhoA-ROCK signaling can reverse the inhibitory effects of these molecules on axon outgrowth and sprouting, which may help promote functional recovery in animal models of CNS injury such as TBI.

Similar to their opposing effects on axon outgrowth, Rac1 typically promotes neuronal survival, whereas RhoA induces apoptotic cell death [20]. RhoA elicits cell death in part by activating ROCK, which phosphorylates and stimulates PTEN, resulting in the inactivation of the pro-survival kinase AKT [19,47]. Thus, increased RhoA-ROCK signaling following TBI likely also contributes to neuronal death, while inhibiting RhoA-ROCK signaling may provide neuroprotection. Indeed, the administration of the ROCK inhibitor fasudil prevents ischemia-induced neuronal apoptosis in rats by maintaining AKT signaling [47]. Inhibition of ROCK with fasudil or Y-27632 also protects neurons from cell death due to excitotoxicity [40,48,49]. Moreover, in a mouse model of brain injury involving hypoxia/reoxygenation, fasudil was found to inhibit ROCK activity in microglia, suppressing inflammatory response and thereby preventing hippocampal neuron loss [50]. TBI also influences the production and survival of adult-born hippocampal neurons, although the exact effect of TBI on adult neurogenesis depends on injury severity [51]. While TBI can increase neurogenesis [52,53,54], it can also induce the selective death of newborn neurons in the dentate gyrus [55,56,57]. Since adult-born neurons are thought to be important for hippocampal-dependent learning and mood regulation [56], enhancing their production and/or survival could aid in the functional recovery from brain injury. Interestingly, fasudil treatment was shown to enhance adult neurogenesis and neuroprotection after hypoxia/reoxygenation injury [58,59], while Y-27632 treatment had little impact on TBI-induced neurogenesis following CCI injury [42]. This disparity could be explained by experimental differences in the type of injury, the specific ROCK inhibitor, and/or the treatment regimen used. Nevertheless, despite these discrepancies, overall the evidence suggests that suppressing cell death may be one mechanism by which RhoA-ROCK inhibition enhances functional recovery from TBI.

Besides promoting axonal outgrowth and/or neuronal survival, targeting the RhoA-ROCK pathway may enhance functional recovery after TBI by preserving synaptic connections, which mediate information flow and storage in the brain [60]. Most excitatory synapses in the mammalian brain are located on actin-rich dendritic spines [61]. Spines range in morphology from long, thin filopodia-like structures to large, mushroom-shaped spines, and their shape is highly correlated with the strength of their associated synapse, with mushroom spines containing the largest, strongest synapses [62]. In response to neural activity, spines rapidly remodel, which is critical for neural circuit development, synaptic plasticity, and processes such as learning and memory [61]. Conversely, aberrant spine morphogenesis, which is a common hallmark of neurodevelopmental, neuropsychiatric, and neurodegenerative disorders, is thought to impair information processing and memory storage [63]. Notably, TBI causes extensive synaptic damage to cortical and hippocampal neurons, as their spines and synapses rapidly degenerate following CCI injury [43,64,65,66,67]. In addition to synapse loss, spines remodel in response to TBI, resulting in a reduction in large mushroom-shaped spines and a corresponding increase in immature filopodia-like structures, compared to sham animals [43,64,68]. Excessive activation of RhoA-ROCK signaling could drive this TBI-induced synaptic remodeling since this pathway is known to promote spine retraction and synapse loss through modulation of the actin cytoskeleton [69] (Figure 1). Indeed, previous work in the retina has shown that ROCK inhibition preserves rod-bipolar synapses after retinal detachment [70]. We investigated this possibility and found that blocking RhoA-ROCK signaling with fasudil treatment prevents TBI-induced pathological spine remodeling in mice subjected to CCI injury [43]. Thus, RhoA-ROCK inhibition may enhance functional recovery after TBI, at least in part, by preventing TBI-induced pathological spine remodeling and synapse loss.

In addition to direct neuronal injury, inflammatory responses involving astrocytes and microglia also contribute to neuronal death and damage after TBI [71]. RhoA-ROCK signaling regulates glial and immune cell functions, and accumulating evidence indicates that activation of this pathway in these cells contributes to neurodegeneration in the CNS [40]. ROCK inhibition reduces reactive gliosis and astrocyte infiltration and increases astrocytic expression of pro-survival genes [40,72,73]. Likewise, ROCK inhibitors decrease microglial inflammatory cytokine release and phagocytosis of neurons, thus promoting neuroprotection [59,72,74,75,76]. However, whether RhoA-ROCK inhibition promotes recovery following TBI by blocking glial cell function remains unclear. For example, Bye et al. found that treatment with the ROCK inhibitor Y-27632, which enhances motor performance in mice following TBI, had little effect on TBI-induced microglial accumulation or astrocytic gliosis [42]. Moreover, we demonstrated that genetically ablating RhoA specifically in post-mitotic forebrain neurons enhances restoration of motor and cognitive function following TBI to a similar extent as fasudil treatment, suggesting that RhoA-ROCK signaling in neurons is primarily responsible for the TBI-induced deficits [43]. Nevertheless, given the important roles RhoA-ROCK signaling plays in regulating astrocyte and microglia function, the effects of RhoA-ROCK inhibition on these cells following TBI requires further consideration. 

## 5. Therapeutic Potential of RhoA-ROCK Inhibitors in the Clinic 

Collectively, the evidence suggests that targeting RhoA-ROCK signaling would be an effective strategy for treating TBI-induced deficits in the clinic. A promising aspect of using pharmacological ROCK inhibitors to treat TBI is that they have a successful track record of use in human patients for the treatment of other disorders, including cerebral vasospasms, glaucoma, and ischemic stroke [40,77,78,79,80,81]. A wide range of small molecule ROCK inhibitors is available, including fasudil, Y-27632, ripasudil, hydroxyfasudil, netarsudil, H-1152, KD-025, and AMA-0076 [30,40,82]. Currently, only fasudil (HA-1077), its derivative ripasudil (K-115), and netarsudil (AR-13324) have been licensed for clinical use, although many other ROCK inhibitors are presently in clinical trials [40,82]. 

Originally designed as an intracellular calcium antagonist, fasudil (Figure 2) was found to be an effective treatment for cerebral vasospasm in an animal model of subarachnoid hemorrhage, and this was confirmed in clinical trials in humans [79,80]. Since its approval for clinical use in 1995, several thousands of people in Japan and China have taken fasudil as a vasodilator to prevent cerebral vasospasm after surgery for subarachnoid hemorrhage and to improve blood flow after acute ischemic stroke [40,78,79,80,81,83]. Fasudil has also been used in clinical trials to treat other conditions such as SCI, ALS, and atherosclerosis [30,84]. Thus, extensive data are available regarding fasudil’s safety and effectiveness. However, despite its clinical success, the use of fasudil faces certain limitations. Although fasudil inhibits both isoforms of ROCK (ROCK1 and ROCK2) more potently than other kinases, its lack of selectivity and low potency (micromolar) are major caveats for its use [40,41,85]. Another long-standing ROCK inhibitor Y-27632 (Figure 2) suffers the same drawbacks [30]. These limitations have encouraged the development of new, more specific, and potent ROCK inhibitors, which are reviewed in more detail elsewhere [39,82,86].

Due to certain adverse effects of ROCK inhibitors when taken systemically, including hypotension, skin reactions, and reversible renal dysfunction [40], the delivery of ROCK inhibitors via local application is gaining favor [82]. The ophthalmology field has led the way with the ROCK inhibitors ripasudil (in Japan) and netarsudil (in the USA) approved as treatments for glaucoma, with several other ROCK inhibitors currently in clinical trials [82,87]. Another interesting approach is to locally inactivate RhoA itself. The exoenzyme C3 transferase is a RhoA antagonist that promotes axon regeneration in models of spinal cord and peripheral nerve injury [88]. A derivative of C3, VX-210 (BA-210/Cethrin), is currently under clinical trials for topical treatment of SCI [88,89], and an initial phase 1/2a study suggested that local application of VX-210 results in significant motor improvement after treatment [90]. However, topical application of drugs to treat CNS disorders such as TBI is more complicated and hence requires further investigation. Another novel approach to avoid the side effects of systemic exposure to ROCK inhibitors is the use of soft drugs. Soft drugs are active compounds that undergo rapid metabolic conversion to an inactive, non-toxic entity once they enter systemic circulation [86]. This approach could maximize exposure to the target organ while minimizing the duration of action of the drug and systemic availability. A variety of soft ROCK inhibitors have been designed, and many pharmaceutical companies are actively investigating their therapeutic potential [82]. For example, topical application of the locally acting ROCK inhibitor AMA-0076 was found to be effective at treating glaucoma without inducing hyperemia [91]. Another interesting line of research that could benefit the treatment of TBI is the development of drugs that selectively target the ROCK2 isoform, which is more abundant in the brain [40]. Unfortunately, due to the high level of structural identity between ROCK1 and ROCK2 and their compensatory functions, designing an isoform-specific inhibitor has been challenging [82]. 

## 6. Concluding Remarks

TBI is a major public health concern that affects millions of people every year. TBI survivors frequently suffer from long-term debilitating physical and emotional deficits. Unfortunately, complete recovery after TBI is often difficult to achieve as current treatments only help manage some of the symptoms. In this review, we have focused on the RhoA-ROCK signaling pathway as a potential target to treat TBI. RhoA signaling is robustly activated after CNS injury, and in mouse models of TBI, blocking RhoA and/or ROCK activity prevents TBI-induced cell death and neuronal damage and improves motor and cognitive performance post-injury. Thus, targeting the RhoA-ROCK pathway appears to be a promising therapeutic approach for treating TBI. Major advances have been made in recent years in the development of novel, potent ROCK and RhoA inhibitors, which are currently in different stages of pre-clinical and clinical testing [82]. However, since many of these drugs are not yet approved for clinical use, repurposing ROCK inhibitors such as fasudil that are already in use for the treatment of other CNS injuries may be the best available option to date to treat TBI. To develop more specific therapeutic targets, it would also be interesting to assess the contribution of various RhoA-GEFs in increasing RhoA activity post-injury. Unfortunately, the role of specific RhoA-GEFs in TBI is currently unclear. While two Rho-family GEFs, GEF-H1 and Cool-2/αPix, have been reported to be activated following TBI [92], the effects of inhibiting these GEFs after TBI remain to be determined. Moreover, although this review focuses exclusively on the RhoA-ROCK pathway as a target for therapeutic intervention, it is likely that combining RhoA/ROCK inhibitors with existing and/or future treatment strategies will result in a greater improvement of motor, behavioral and cognitive symptoms in individuals suffering from a TBI.

## References

[1] Taylor C.A., Bell J.M., Breiding M.J., Xu L. (2017). Traumatic Brain Injury-Related Emergency Department Visits, Hospitalizations, and Deaths—United States, 2007 and 2013. MMWR Surveill. Summ..

[2] Whelan-Goodinson R., Ponsford J., Johnston L., Grant F. (2009). Psychiatric disorders following traumatic brain injury: Their nature and frequency. J. Head Trauma Rehabil..

[3] Barker-Collo S., Theadom A., Jones K., Starkey N., Kahan M., Feigin V. (2018). Depression and anxiety across the first 4 years after mild traumatic brain injury: Findings from a community-based study. Brain Inj..

[4] Clark M., Guskiewicz K., Laskowitz D., Grant G. (2016). Sport-Related Traumatic Brain Injury. Translational Research in Traumatic Brain Injury.

[5] Maroon J.C., Winkelman R., Bost J., Amos A., Mathyssek C., Miele V. (2015). Chronic traumatic encephalopathy in contact sports: A systematic review of all reported pathological cases. PLoS ONE.

[6] Hoge C.W., Castro C.A., Messer S.C., McGurk D., Cotting D.I., Koffman R.L. (2008). Combat duty in Iraq and Afghanistan, mental health problems and barriers to care. US Army Med. Dep. J..

[7] Schneiderman A.I., Braver E.R., Kang H.K. (2008). Understanding sequelae of injury mechanisms and mild traumatic brain injury incurred during the conflicts in Iraq and Afghanistan: Persistent postconcussive symptoms and posttraumatic stress disorder. Am. J. Epidemiol..

[8] Pearn M.L., Niesman I.R., Egawa J., Sawada A., Almenar-Queralt A., Shah S.B., Duckworth J.L., Head B.P. (2017). Pathophysiology Associated with Traumatic Brain Injury: Current Treatments and Potential Novel Therapeutics. Cell. Mol. Neurobiol..

[9] Hodge R.G., Ridley A.J. (2016). Regulating Rho GTPases and their regulators. Nat. Rev. Mol. Cell Biol..

[10] Cherfils J., Zeghouf M. (2013). Regulation of small GTPases by GEFs, GAPs, and GDIs. Physiol. Rev..

[11] Duman J.G., Mulherkar S., Tu Y.K., Cheng J.X., Tolias K.F. (2015). Mechanisms for spatiotemporal regulation of Rho-GTPase signaling at synapses. Neurosci. Lett..

[12] Leung T., Manser E., Tan L., Lim L. (1995). A novel serine/threonine kinase binding the Ras-related RhoA GTPase which translocates the kinase to peripheral membranes. J. Biol. Chem..

[13] Matsui T., Amano M., Yamamoto T., Chihara K., Nakafuku M., Ito M., Nakano T., Okawa K., Iwamatsu A., Kaibuchi K. (1996). Rho-associated kinase, a novel serine/threonine kinase, as a putative target for small GTP binding protein Rho. EMBO J..

[14] Amano M., Nakayama M., Kaibuchi K. (2010). Rho-kinase/ROCK: A key regulator of the cytoskeleton and cell polarity. Cytoskeleton.

[15] Sit S.T., Manser E. (2011). Rho GTPases and their role in organizing the actin cytoskeleton. J. Cell Sci..

[16] Kanellos G., Frame M.C. (2016). Cellular functions of the ADF/cofilin family at a glance. J. Cell Sci..

[17] Maekawa M., Ishizaki T., Boku S., Watanabe N., Fujita A., Iwamatsu A., Obinata T., Ohashi K., Mizuno K., Narumiya S. (1999). Signaling from Rho to the actin cytoskeleton through protein kinases ROCK and LIM-kinase. Science.

[18] Arimura N., Menager C., Fukata Y., Kaibuchi K. (2004). Role of CRMP-2 in neuronal polarity. J. Neurobiol..

[19] Li Z., Dong X., Wang Z., Liu W., Deng N., Ding Y., Tang L., Hla T., Zeng R., Li L. (2005). Regulation of PTEN by Rho small GTPases. Nat. Cell Biol..

[20] Stankiewicz T.R., Linseman D.A. (2014). Rho family GTPases: Key players in neuronal development, neuronal survival, and neurodegeneration. Front. Cell. Neurosci..

[21] Nakayama A.Y., Harms M.B., Luo L. (2000). Small GTPases Rac and Rho in the maintenance of dendritic spines and branches in hippocampal pyramidal neurons. J. Neurosci..

[22] Govek E., Hatten M., Van Aelst L. (2011). The role of Rho GTPase proteins in CNS neuronal migration. Dev. Neurobiol..

[23] Cappello S., Böhringer C., Bergami M., Conzelmann K., Ghanem A., Tomassy G., Arlotta P., Mainardi M., Allegra M., Caleo M. (2012). A radial glia-specific role of RhoA in double cortex formation. Neuron.

[24] Herzog D., Loetscher P., van Hengel J., Knüsel S., Brakebusch C., Taylor V., Suter U., Relvas J.B. (2011). The small GTPase RhoA is required to maintain spinal cord neuroepithelium organization and the neural stem cell pool. J. Neurosci..

[25] Katayama K., Leslie J.R., Lang R.A., Zheng Y., Yoshida Y. (2012). Left-right locomotor circuitry depends on RhoA-driven organization of the neuroepithelium in the developing spinal cord. J. Neurosci..

[26] Mulherkar S., Liu F., Chen Q., Narayanan A., Couvillon A.D., Shine H.D., Tolias K.F. (2013). The small GTPase RhoA is required for proper locomotor circuit assembly. PLoS ONE.

[27] Mulherkar S., Uddin M.D., Couvillon A.D., Sillitoe R.V., Tolias K.F. (2014). The small GTPases RhoA and Rac1 regulate cerebellar development by controlling cell morphogenesis, migration and foliation. Dev. Biol..

[28] Tolias K.F., Duman J.G., Um K. (2011). Control of synapse development and plasticity by Rho GTPase regulatory proteins. Prog. Neurobiol..

[29] Fujita Y., Yamashita T. (2014). Axon growth inhibition by RhoA/ROCK in the central nervous system. Front. Neurosci..

[30] Loirand G. (2015). Rho Kinases in Health and Disease: From Basic Science to Translational Research. Pharmacol. Rev..

[31] Forgione N., Fehlings M.G. (2014). Rho-ROCK inhibition in the treatment of spinal cord injury. World Neurosurg..

[32] Dubreuil C.I., Marklund N., Deschamps K., McIntosh T.K., McKerracher L. (2006). Activation of Rho after traumatic brain injury and seizure in rats. Exp. Neurol..

[33] Dubreuil C.I., Winton M.J., McKerracher L. (2003). Rho activation patterns after spinal cord injury and the role of activated Rho in apoptosis in the central nervous system. J. Cell Biol..

[34] Erschbamer M.K., Hofstetter C.P., Olson L. (2005). RhoA, RhoB, RhoC, Rac1, Cdc42, and Tc10 mRNA levels in spinal cord, sensory ganglia, and corticospinal tract neurons and long-lasting specific changes following spinal cord injury. J. Comp. Neurol..

[35] Wang J., Zarbin M., Sugino I., Whitehead I., Townes-Anderson E. (2016). RhoA Signaling and Synaptic Damage Occur Within Hours in a Live Pig Model of CNS Injury, Retinal Detachment. Investig. Ophthalmol. Vis. Sci..

[36] Wang W., Townes-Anderson E. (2015). LIM Kinase, a Newly Identified Regulator of Presynaptic Remodeling by Rod Photoreceptors After Injury. Investig. Ophthalmol. Vis. Sci..

[37] Brabeck C., Beschorner R., Conrad S., Mittelbronn M., Bekure K., Meyermann R., Schluesener H.J., Schwab J.M. (2004). Lesional expression of RhoA and RhoB following traumatic brain injury in humans. J. Neurotrauma.

[38] Chen M., Liu A., Ouyang Y., Huang Y., Chao X., Pi R. (2013). Fasudil and its analogs: A new powerful weapon in the long war against central nervous system disorders?. Expert Opin. Investig. Drugs.

[39] Feng Y., LoGrasso P.V., Defert O., Li R. (2016). Rho Kinase (ROCK) Inhibitors and Their Therapeutic Potential. J. Med. Chem..

[40] Koch J.C., Tatenhorst L., Roser A.E., Saal K.A., Tonges L., Lingor P. (2018). ROCK inhibition in models of neurodegeneration and its potential for clinical translation. Pharmacol. Ther..

[41] Olson M.F. (2008). Applications for ROCK kinase inhibition. Curr. Opin. Cell Biol..

[42] Bye N., Christie K.J., Turbic A., Basrai H.S., Turnley A.M. (2016). Rho kinase inhibition following traumatic brain injury in mice promotes functional improvement and acute neuron survival but has little effect on neurogenesis, glial responses or neuroinflammation. Exp. Neurol..

[43] Mulherkar S., Firozi K., Huang W., Uddin M.D., Grill R.J., Costa-Mattioli M., Robertson C., Tolias K.F. (2017). RhoA-ROCK Inhibition Reverses Synaptic Remodeling and Motor and Cognitive Deficits Caused by Traumatic Brain Injury. Sci. Rep..

[44] McKerracher L., Rosen K.M. (2015). MAG, myelin and overcoming growth inhibition in the CNS. Front. Mol. Neurosci..

[45] Niederöst B., Oertle T., Fritsche J., McKinney R.A., Bandtlow C.E. (2002). Nogo-A and myelin-associated glycoprotein mediate neurite growth inhibition by antagonistic regulation of RhoA and Rac1. J. Neurosci..

[46] Schweigreiter R., Walmsley A.R., Niederöst B., Zimmermann D.R., Oertle T., Casademut E., Frentzel S., Dechant G., Mir A., Bandtlow C.E. (2004). Versican V2 and the central inhibitory domain of Nogo-A inhibit neurite growth via p75NTR/NgR-independent pathways that converge at RhoA. Mol. Cell. Neurosci..

[47] Wu J., Li J., Hu H., Liu P., Fang Y., Wu D. (2012). Rho-kinase inhibitor, fasudil, prevents neuronal apoptosis via the Akt activation and PTEN inactivation in the ischemic penumbra of rat brain. Cell. Mol. Neurobiol..

[48] Jeon B.T., Jeong E.A., Park S.Y., Son H., Shin H.J., Lee D.H., Kim H.J., Kang S.S., Cho G.J., Choi W.S. (2013). The Rho-kinase (ROCK) inhibitor Y-27632 protects against excitotoxicity-induced neuronal death in vivo and in vitro. Neurotox. Res..

[49] Kitaoka Y., Kitaoka Y., Kumai T., Lam T.T., Kuribayashi K., Isenoumi K., Munemasa Y., Motoki M., Kobayashi S., Ueno S. (2004). Involvement of RhoA and possible neuroprotective effect of fasudil, a Rho kinase inhibitor, in NMDA-induced neurotoxicity in the rat retina. Brain Res..

[50] Ding J., Li Q.Y., Wang X., Sun C.H., Lu C.Z., Xiao B.G. (2010). Fasudil protects hippocampal neurons against hypoxia-reoxygenation injury by suppressing microglial inflammatory responses in mice. J. Neurochem..

[51] Wang X., Gao X., Michalski S., Zhao S., Chen J. (2016). Traumatic Brain Injury Severity Affects Neurogenesis in Adult Mouse Hippocampus. J. Neurotrauma.

[52] Chirumamilla S., Sun D., Bullock M.R., Colello R.J. (2002). Traumatic brain injury induced cell proliferation in the adult mammalian central nervous system. J. Neurotrauma.

[53] Dash P.K., Mach S.A., Moore A.N. (2001). Enhanced neurogenesis in the rodent hippocampus following traumatic brain injury. J. Neurosci. Res..

[54] Urrea C., Castellanos D.A., Sagen J., Tsoulfas P., Bramlett H.M., Dietrich W.D. (2007). Widespread cellular proliferation and focal neurogenesis after traumatic brain injury in the rat. Restor. Neurol. Neurosci..

[55] Gao X., Deng-Bryant Y., Cho W., Carrico K.M., Hall E.D., Chen J. (2008). Selective death of newborn neurons in hippocampal dentate gyrus following moderate experimental traumatic brain injury. J. Neurosci. Res..

[56] Ngwenya L.B., Danzer S.C. (2018). Impact of Traumatic Brain Injury on Neurogenesis. Front. Neurosci..

[57] Zhou H., Chen L., Gao X., Luo B., Chen J. (2012). Moderate traumatic brain injury triggers rapid necrotic death of immature neurons in the hippocampus. J. Neuropathol. Exp. Neurol..

[58] Ding J., Li Q.Y., Yu J.Z., Wang X., Sun C.H., Lu C.Z., Xiao B.G. (2010). Fasudil, a Rho kinase inhibitor, drives mobilization of adult neural stem cells after hypoxia/reoxygenation injury in mice. Mol. Cell. Neurosci..

[59] Ding J., Yu J.Z., Li Q.Y., Wang X., Lu C.Z., Xiao B.G. (2009). Rho kinase inhibitor Fasudil induces neuroprotection and neurogenesis partially through astrocyte-derived G-CSF. Brain Behav. Immun..

[60] Wen Z., Li D., Shen M., Chen G. (2017). Therapeutic Potentials of Synapses after Traumatic Brain Injury: A Comprehensive Review. Neural Plast..

[61] Spence E.F., Soderling S.H. (2015). Actin Out: Regulation of the Synaptic Cytoskeleton. J. Biol. Chem..

[62] Kasai H., Fukuda M., Watanabe S., Hayashi-Takagi A., Noguchi J. (2010). Structural dynamics of dendritic spines in memory and cognition. Trends Neurosci..

[63] Van Spronsen M., Hoogenraad C.C. (2010). Synapse pathology in psychiatric and neurologic disease. Curr. Neurol. Neurosci. Rep..

[64] Gao X., Chen J. (2011). Mild traumatic brain injury results in extensive neuronal degeneration in the cerebral cortex. J. Neuropathol. Exp. Neurol..

[65] Perez E.J., Cepero M.L., Perez S.U., Coyle J.T., Sick T.J., Liebl D.J. (2016). EphB3 signaling propagates synaptic dysfunction in the traumatic injured brain. Neurobiol. Dis..

[66] Pijet B., Stefaniuk M., Kaczmarek L. (2019). MMP-9 Contributes to Dendritic Spine Remodeling Following Traumatic Brain Injury. Neural Plast..

[67] Winston C.N., Chellappa D., Wilkins T., Barton D.J., Washington P.M., Loane D.J., Zapple D.N., Burns M.P. (2013). Controlled cortical impact results in an extensive loss of dendritic spines that is not mediated by injury-induced amyloid-beta accumulation. J. Neurotrauma.

[68] Gao X., Deng P., Xu Z.C., Chen J. (2011). Moderate traumatic brain injury causes acute dendritic and synaptic degeneration in the hippocampal dentate gyrus. PLoS ONE.

[69] Lai K.O., Ip N.Y. (2013). Structural plasticity of dendritic spines: The underlying mechanisms and its dysregulation in brain disorders. Biochim. Biophys. Acta.

[70] Townes-Anderson E., Wang J., Halasz E., Sugino I., Pitler A., Whitehead I., Zarbin M. (2017). Fasudil, a Clinically Used ROCK Inhibitor, Stabilizes Rod Photoreceptor Synapses after Retinal Detachment. Transl. Vis. Sci. Technol..

[71] Karve I.P., Taylor J.M., Crack P.J. (2016). The contribution of astrocytes and microglia to traumatic brain injury. Br. J. Pharmacol..

[72] Lau C.L., Perreau V.M., Chen M.J., Cate H.S., Merlo D., Cheung N.S., O’Shea R.D., Beart P.M. (2012). Transcriptomic profiling of astrocytes treated with the Rho kinase inhibitor fasudil reveals cytoskeletal and pro-survival responses. J. Cell. Physiol..

[73] Tura A., Schuettauf F., Monnier P.P., Bartz-Schmidt K.U., Henke-Fahle S. (2009). Efficacy of Rho-kinase inhibition in promoting cell survival and reducing reactive gliosis in the rodent retina. Investig. Ophthalmol. Vis. Sci..

[74] Barcia C., Ros C.M., Annese V., Carrillo-de Sauvage M.A., Ros-Bernal F., Gomez A., Yuste J.E., Campuzano C.M., de Pablos V., Fernandez-Villalba E. (2012). ROCK/Cdc42-mediated microglial motility and gliapse formation lead to phagocytosis of degenerating dopaminergic neurons in vivo. Sci. Rep..

[75] Holtje M., Hoffmann A., Hofmann F., Mucke C., Grosse G., Van Rooijen N., Kettenmann H., Just I., Ahnert-Hilger G. (2005). Role of Rho GTPase in astrocyte morphology and migratory response during in vitro wound healing. J. Neurochem..

[76] Zhang H., Li Y., Yu J., Guo M., Meng J., Liu C., Xie Y., Feng L., Xiao B., Ma C. (2013). Rho kinase inhibitor fasudil regulates microglia polarization and function. Neuroimmunomodulation.

[77] Honjo M., Tanihara H. (2018). Impact of the clinical use of ROCK inhibitor on the pathogenesis and treatment of glaucoma. Jpn. J. Ophthalmol..

[78] Shibuya M., Hirai S., Seto M., Satoh S., Ohtomo E., Fasudil Ischemic Stroke Study Group (2005). Effects of fasudil in acute ischemic stroke: Results of a prospective placebo-controlled double-blind trial. J. Neurol. Sci..

[79] Shibuya M., Suzuki Y., Sugita K., Saito I., Sasaki T., Takakura K., Nagata I., Kikuchi H., Takemae T., Hidaka H. (1992). Effect of AT877 on cerebral vasospasm after aneurysmal subarachnoid hemorrhage. Results of a prospective placebo-controlled double-blind trial. J. Neurosurg..

[80] Zhao J., Zhou D., Guo J., Ren Z., Zhou L., Wang S., Xu B., Wang R. (2006). Effect of fasudil hydrochloride, a protein kinase inhibitor, on cerebral vasospasm and delayed cerebral ischemic symptoms after aneurysmal subarachnoid hemorrhage. Neurol. Med. Chir..

[81] Zhao J., Zhou D., Guo J., Ren Z., Zhou L., Wang S., Zhang Y., Xu B., Zhao K., Wang R. (2011). Efficacy and safety of fasudil in patients with subarachnoid hemorrhage: Final results of a randomized trial of fasudil versus nimodipine. Neurol. Med. Chir..

[82] Defert O., Boland S. (2017). Rho kinase inhibitors: A patent review (2014–2016). Expert Opin. Ther. Pat..

[83] Suzuki Y., Shibuya M., Satoh S., Sugimoto Y., Takakura K. (2007). A postmarketing surveillance study of fasudil treatment after aneurysmal subarachnoid hemorrhage. Surg. Neurol..

[84] Lingor P., Weber M., Camu W., Friede T., Hilgers R., Leha A., Neuwirth C., Gunther R., Benatar M., Kuzma-Kozakiewicz M. (2019). ROCK-ALS: Protocol for a Randomized, Placebo-Controlled, Double-Blind Phase IIa Trial of Safety, Tolerability and Efficacy of the Rho Kinase (ROCK) Inhibitor Fasudil in Amyotrophic Lateral Sclerosis. Front. Neurol..

[85] Ono-Saito N., Niki I., Hidaka H. (1999). H-series protein kinase inhibitors and potential clinical applications. Pharmacol. Ther..

[86] Boland S., Bourin A., Alen J., Geraets J., Schroeders P., Castermans K., Kindt N., Boumans N., Panitti L., Fransen S. (2015). Design, synthesis, and biological evaluation of novel, highly active soft ROCK inhibitors. J. Med. Chem..

[87] Kish T. (2018). Old and New Drug Classes Expanding to Include Glaucoma Treatments. Pharm. Ther..

[88] Ng Y., Lee Y. (2019). Traumatic Brain Injuries: Pathophysiology and Potential Therapeutic Targets. Front. Cell. Neurosci..

[89] Fehlings M.G., Kim K.D., Aarabi B., Rizzo M., Bond L.M., McKerracher L., Vaccaro A.R., Okonkwo D.O. (2018). Rho Inhibitor VX-210 in Acute Traumatic Subaxial Cervical Spinal Cord Injury: Design of the SPinal Cord Injury Rho INhibition InvestiGation (SPRING) Clinical Trial. J. Neurotrauma.

[90] Fehlings M.G., Theodore N., Harrop J., Maurais G., Kuntz C., Shaffrey C.I., Kwon B.K., Chapman J., Yee A., Tighe A. (2011). A phase I/IIa clinical trial of a recombinant Rho protein antagonist in acute spinal cord injury. J. Neurotrauma.

[91] Van de Velde S., Van Bergen T., Sijnave D., Hollanders K., Castermans K., Defert O., Leysen D., Vandewalle E., Moons L., Stalmans I. (2014). AMA0076, a novel, locally acting Rho kinase inhibitor, potently lowers intraocular pressure in New Zealand white rabbits with minimal hyperemia. Investig. Ophthalmol. Vis. Sci..

[92] Sabirzhanova I., Liu C., Zhao J., Bramlett H., Dalton D., Hu B. (2013). Changes in GEF-H1 Pathways after Traumatic Brain Injury. J. Neurotrauma.

